# Recent Progress in Fluorescent Imaging Probes

**DOI:** 10.3390/s150924374

**Published:** 2015-09-22

**Authors:** Yen Leng Pak, K. M. K. Swamy, Juyoung Yoon

**Affiliations:** Department of Chemistry and Nano Sciences, Ewha Womans University, Seoul 120-750, Korea; E-Mails: xiaobai080488@yahoo.com (Y.L.P.); kmkswamy@yahoo.com (K.M.K.S.)

**Keywords:** fluorescent probes, imaging probes, fluorescent chemosensors, fluorescent chemosensors for biothiols, fluorescent chemosensors for ROS, fluorescent chemosensors for metal ions

## Abstract

Due to the simplicity and low detection limit, especially the bioimaging ability for cells, fluorescence probes serve as unique detection methods. With the aid of molecular recognition and specific organic reactions, research on fluorescent imaging probes has blossomed during the last decade. Especially, reaction based fluorescent probes have been proven to be highly selective for specific analytes. This review highlights our recent progress on fluorescent imaging probes for biologically important species, such as biothiols, reactive oxygen species, reactive nitrogen species, metal ions including Zn^2+^, Hg^2+^, Cu^2+^ and Au^3+^, and anions including cyanide and adenosine triphosphate (ATP).

## 1. Introduction

The recognition of biologically and environmentally important species in addition to imaging them has been an important research task in recent years [1,2,3]. Compared to other analytical tools, fluorescent probes have several merits, including simplicity, low detection limit, and most importantly, cell-imaging with the aid of confocal microscopy [4,5,6,7,8,9,10]. Recent developments on near IR (NIR) fluorescent probes and two-photon fluorescent probes have enabled the visualization of biologically important analytes in the mouse and in tissues [11,12,13].

Traditional approaches using molecular recognition and host–guest chemistry have been adopted to design fluorescent chemosensors for many years [14]. de Silva [15], Czarnik [16,17] *et al.* reported pioneering works in this regards. Earlier works on fluorescent chemosensors further extended their scopes for various biologically important analytes. The most dramatic change in this field was the appearance of reaction-based fluorescent probes, so-called chemodosimeters [18,19,20], which react with specific analytes, resulting in irreversible optical changes, yet, with usually better selectivity than those originating from host–guest chemistry.

In this review, we will cover our recent contributions to this exciting topic. This review highlights the recent progress on fluorescent imaging probes for biologically important species, such as biothiols, reactive oxygen species, reactive nitrogen species, metal ions including Zn^2+^, Hg^2+^, Cu^2+^ and Au^3+^, and anions including cyanide and ATP.

## 2. Fluorescent Probes on Biologically Important Species

### 2.1. Fluorescent Probes for Biothiols

Biothiols, such as cysteine (Cys), homocysteine (Hcy) and glutathione (GSH), play key roles in physiological systems. It is known that abnormal intracellular thiols are closely related to various health problems. Accordingly, fluorescent probes for these biothiols have attracted great attention in recent years [21].

**Figure 1:**
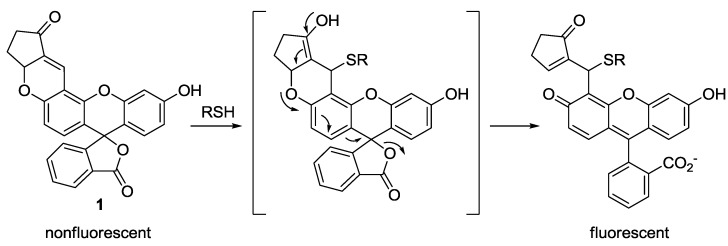
Reaction scheme for biothiol selective probe **1**.

A few years ago, our group introduced fluorescein-based probe **1** as a fluorescent probe for biological thiols (Figure 1) [22]. As shown in Figure 1, the spiro lactone ring opening occurred upon the addition of biothiols (GSH, Cys and Hcy) to the α,β-unsaturated ketone, resulting in fluorescence enhancement (λ_max_ = 520 nm) in HEPES buffer (20 mM, pH 7.4, 1% CH_3_CN). To monitor thiols in living cells and organisms, murine P19 embryonic carcinoma cells and a three-day-old zebrafish were incubated with **1**. Strong fluorescence enhancement was observed inside the cells and zebrafish. When zebrafish and cells were pretreated with a trapping reagent of thiols, *N*-methylmaleimide (NMM), significant fluorescence quenching was observed, which indicates that probe **1** images biothiols, especially GSH, present in the cells or zebra fish.

Non-selective biothiol probes can usually image GSH, the most abundant biothiol in cells. For Cys and Hcy, selective recognition of these biothiols is certainly required, which is quite a challenging task due to the structural similarity of Cys and Hcy. In 2011, a pioneering work was reported by Strongin’s group, in which an α,β-unsaturated carbonyl recognition unit was first utilized to discriminate Cys from Hcy with a kinetically favored 7-membered ring formation [23].

**Figure 2:**
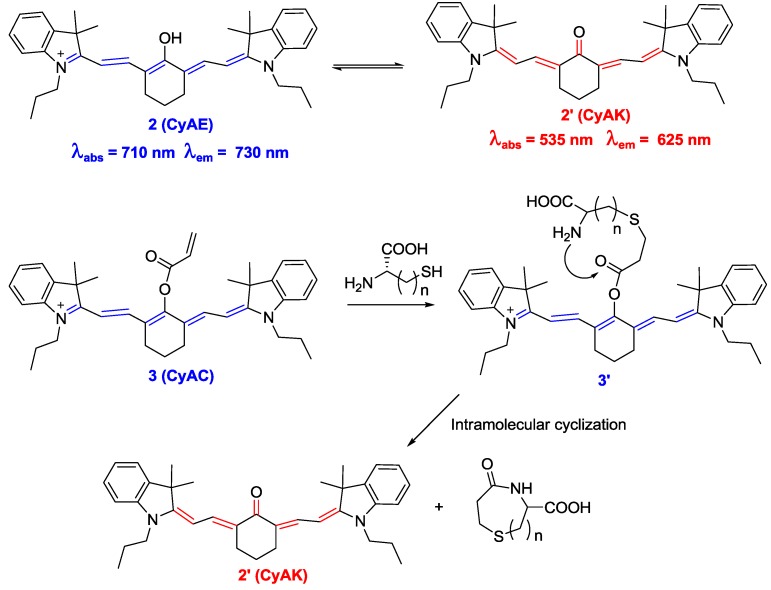
Comparison between CyAE (**2**) and CyAK (**2′**) and reaction scheme for Cys selective probe **3** (CyAC).

We recently applied Strongin’s strategy to a new near-infrared (NIR) cyanine-based probe **3** (CyAC), which can selectively sense Cys over Hcy and GSH (Figure 2) [24]. In this study, we proposed a new strategy to induce distinct optical changes in the cyanine system. As shown in Figure 2, the absorption and emission maximums of hydroxy cyanine CyAE**2** and its keto form **2′** can be distinctively different with the modulation of polymethine π-electron systems. Accordingly, the title probe **3** containing an acrylate group as a trigger moiety for Cys can show similar large shifts in its absorption and emission spectra (from 770 to 515 nm for absorption and from 780 to 570 nm for emission) after adduct-cyclization reaction with Cys. Probe **3** was applied to the biological imaging of Cys in MCF-7 cells grown in glucose-free Dulbecco’s modified Eagle medium. During glucose deprivation, the intracellular Cys level is known to be significantly increased. Large fluorescence enhancement at 590 nm and a dramatic fluorescence decrease in the NIR region (760–855 nm) was observed under this condition.

**Figure 3:**
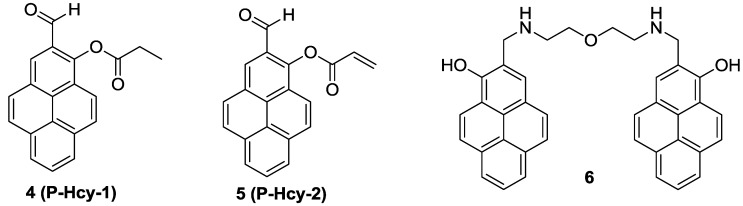
Structures of biothiol probes **4**–**6**.

On the other hand, new pyrene-based fluorescence probes, **4** (**P-Hcy-1**) and **5** (**P-Hcy-2**), were reported as Hcy selective probes by our group (Figure 3) [25]. **P-Hcy-1** and **P-Hcy-2** displayed selective fluorescence enhancements at 450 nm with Hcy in HEPES containing 10% DMSO (0.01 M, pH 7.4). In both cases, the thiazinane heterocyclic ring formation of the aldehyde group turned out to be the reason for their Hcy selective fluorescence responses. The detection limits of **P-Hcy-1** and **P-Hcy-2** were calculated as 1.94 × 10^−6^ M and 1.44 × 10^−7^ M, respectively. These Hcy probes were successfully applied to detect Hcy selectively in mammalian cells.

Among the various approaches to design biothiol selective probes, fluorescence enhancement of Cu^2+^ complexes has been utilized for biothiol imaging. A new bis-pyrene derivative **6** was synthesized and a large fluorescence quenching effect was observed with Cu^2+^ at pH 7.4 among the various metal ions (Figure 3) [26]. The addition of biothiols successfully revived the fluorescence of the **6**-Cu^2+^ ensemble, which was attributed to the displacement of the ligand to the biothiols for Cu^2+^. The addition of GSH, Cys or Hcy effectively induced fluorescence enhancement at 450 nm. The detection limit of this **6**-Cu^2+^ ensemble for GSH was reported to be 0.16 µM. The Cu^2+^ ensemble could successfully image endogenous GSH in live cells and with the aid of two-photon microscopy (TPM), the **6**-Cu^2+^ ensemble could also image GSH in living tissues.

**Figure 4:**
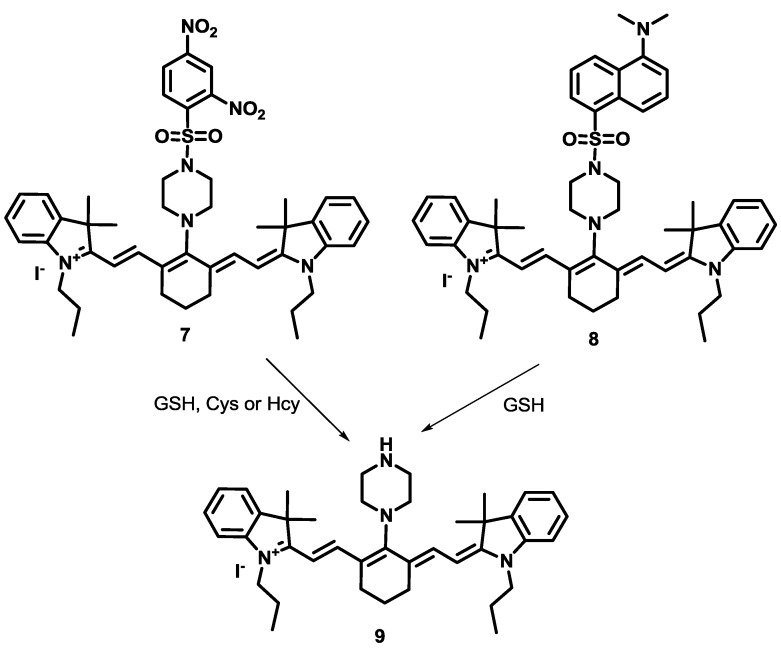
Reaction scheme for biothiol selective probe **7** and GSH selective probe **8**.

**Figure 5:**
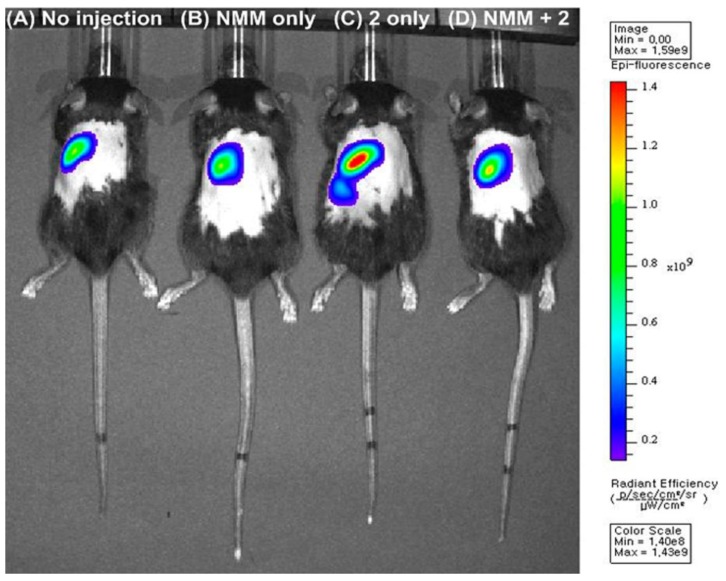
*In vivo* images of a mouse injected with probe **8** (50 µM) or NMM (20 mM) intravenously for 20 min. Fluorescence images of: (**A**) the mouse not injected with probe **8** (No injection); (**B**) the mouse injected with NMM (NMM only); (**C**) the mouse injected with probe **8** (**8** only); (**D**) the mouse injected with probe **8** after pre-injection with NMM. (Reprinted from reference [27]).

Compared to Cys and Hcy selective probes, GSH selective fluorescent imaging probes are still relatively rare. Recently, a new approach to designing GSH selective probes was reported by our group. We synthesized two cyanine derivatives containing a 2,4-dinitrobenzene sulfonamide group (**7**) and a 5-dimethylaminonaphthyl sulfonamide group (**8**), respectively, as NIR probes for biothiols (Figure 4) [27]. Probe **7** showed large fluorescence enhancements at 736 nm with GSH, Cys and Hcy in HEPES buffer (10 mM, pH = 7.4) containing 10% DMSO. On the contrary, probe **8** displayed selective turn-on fluorescence with GSH. After reaction with biothiols, product **9** was confirmed by NMR and mass spectroscopy. These fluorescence changes with biothiols were further confirmed in HeLa cells. Probe **8** could image the GSH present in HeLa cells and no fluorescence was observed upon treatment with the thiol blocking agent, *N*-methylmaleimide (NMM). We also demonstrated that probe **9** can be used to monitor the intracellular levels of GSH modulated by H_2_O_2_ or lipopolysaccharide (LPS) treatment. NIR probe **8** was further applied to monitor GSH in a mouse model (Figure 5), especially in liver, kidney, lung, and spleen tissues. It is reported that overdose of the painkiller, acetaminophen, can cause severe liver damage and a depletion of GSH in liver and kidney cells. The depletion of GSH in mouse tissue cells was successfully monitored using probe **8**.

**Figure 6:**
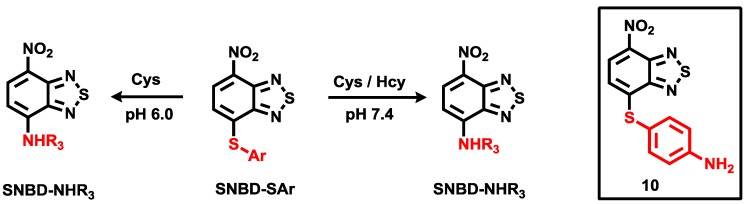
Structure of probe **10** and its reaction scheme with Cys and Hcy.

For Cys and Hcy selective probes, we developed the aryl-thioether substituted nitrobenzothiadiazole **10** (Figure 6) [28]. Only Cys and Hcy induced fluorescence enhancement (λ_max_ = 535 nm) at pH 7.4. The proposed reaction scheme with Cys and Hcy is illustrated in Figure 6. We also reported that probe **10** could image these biothiol species in live cells. It is known that the nucleophilicity of Cys (*pK_a_* 8.53) is better than that of Hcy (*pK_a_* 10.00). In addition, we expect that the *p-*amino group in probe **10** can manipulate the aryl substitution selectivity at lower pH. Indeed, we observed nice selectivity for Cys over Hcy in a citric acid-Na_2_HPO_4_ (0.01 M, pH 6.0) solution containing 1% DMSO.

### 2.2. Fluorescent Imaging Probes for Reactive Oxygen Species (ROS) and Reactive Nitrogen Species (RNS)

Reactive oxygen species (ROS) and reactive nitrogen species (RNS) are active targets for fluorescent probes due to their significance in human health and disease [29]. We also contributed to the development of ROS and RNS selective fluorescent probes, especially for hypochlorous acid (HOCl), hydrogen peroxide (H_2_O_2_) and peroxynitrite (ONOO^−^).

**Figure 7:**
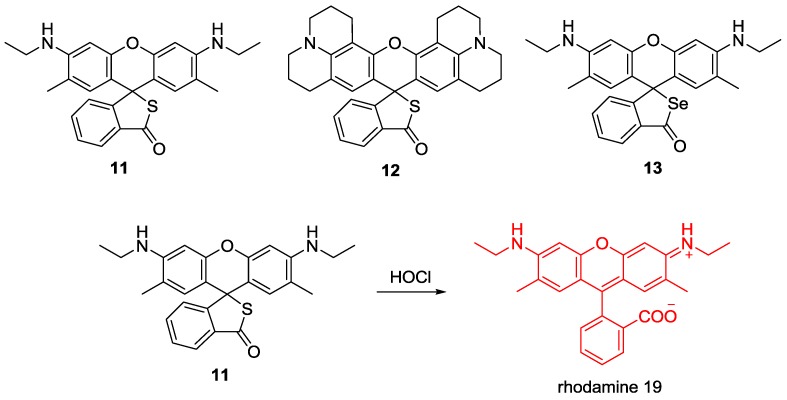
Structures of HOCl selective fluorescent probes **11**–**13** and reaction scheme for **11** with HOCl.

Novel rhodamine derivatives **11**–**13** were synthesized as fluorescent probes for HOCl (Figure 7) [30]. Fluorescent increase upon the addition of HOCl was attributed to the ring-opening process through sulfur/selenium oxidation as shown in Figure 7. Probes **11** and **12** showed highly selective fluorescence enhancement with HOCl among the various ROS, such as H_2_O_2_, NO•, •OH, ROO•, ONOO^−^, ^1^O_2_, and •O_2_^−^ in KH_2_PO_4_ buffer (pH 5.5, 1% CH_3_CN). On the other hand, probe **13** displayed a lower selectivity for HOCl, probably due to the higher susceptibility to selenolactone by oxidants.The detection limits of HOCl probes **11**–**13** were calculated as 0.4 μM, 0.6 μM and 2 μM, respectively. Furthermore, probe **11** could image bacteria-mediated microbicidal HOCl production in the mucosal epithelia in fruit fly.

**Figure 8:**
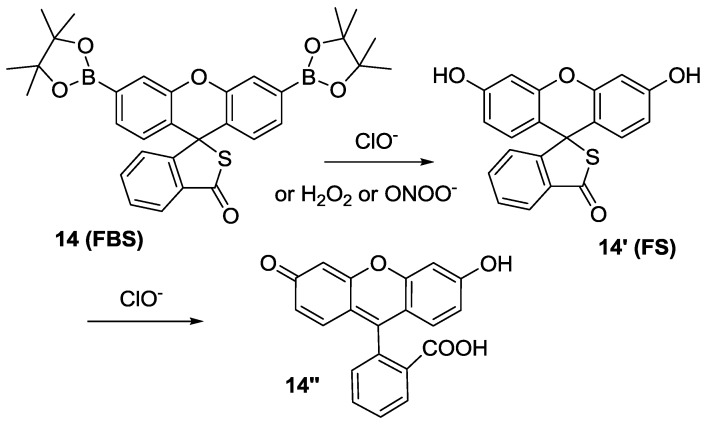
Reaction scheme for HOCl selective fluorescent probe **14** (**FBS**).

We extended this design concept to the so-called “dual-lock” fluorescent probe **14** (**FBS**) for HOCl (Figure 8) [31], in which two reacting groups, such as aryl boronate and thiolactone, are introduced. Among the various ROS, H_2_O_2_ and ONOO^−^ could convert arylboronates to phenol to yield **14′**, which is still non-fluorescent. On the contrary, only HOCl can react with both arylboronates and thiolactone to give fluorescein (**14″**) resulting in green fluorescence. Another advantage of **FBS** would be its large pH window between pH 5.5 and pH 9.3. **FBS** was further applied to detect physiological HOCl production *in vivo*. For this purpose, we chose the *drosophila* gut system, a well-known HOCl producing organ. FBS could successfully image bacterial-induced HOCl production *in situ* when bacterial extracts were administered to the flies via oral ingestion.

**Figure 9:**
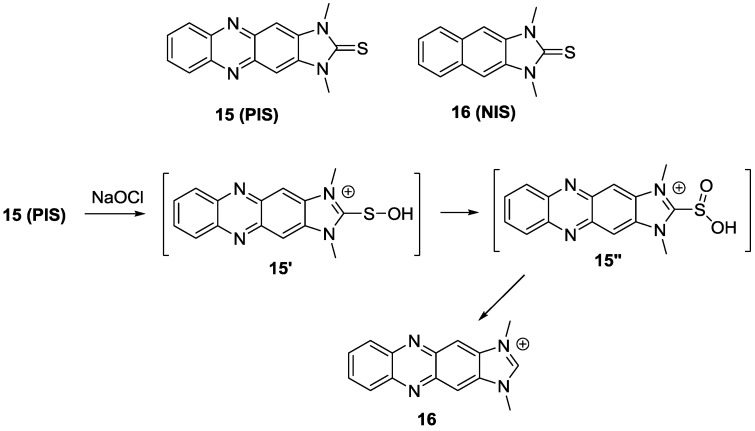
Structures of **15** (**PIS**) and **16** (**NIS**) and reaction scheme of **15** (**PIS**) with HOCl.

**Figure 10:**
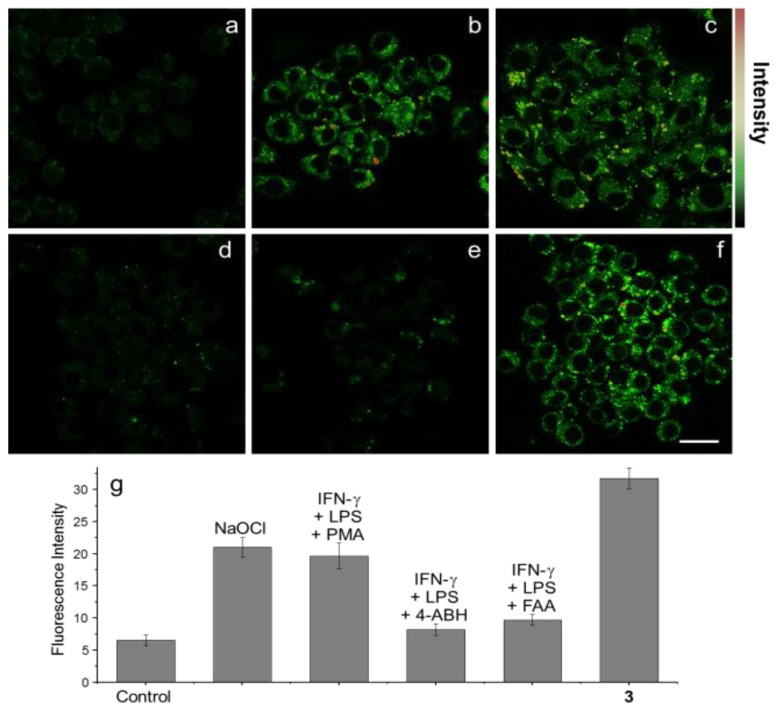
TPM images of (**a**–**e**) **PIS** and (**f**) **16** (10 μM, ρ_DMF_ = 0.5%) labeled RAW 264.7 cells. (**a**) Control image; (**b**) Cells pretreated with NaOCl (200 μM) for 30 min and then incubated with **PIS**; (**c**) Cells pretreated with LPS (100 ng/mL) for 16 h, IFN-γ (400 U/mL) for 4 h, and PMA (10 nM) for 30 min and then with **PIS**; (**d**) Cells pretreated with LPS, IFN-γ, and 4-ABAH (50 μM) for 4 h and then incubated with **PIS**; (**e**) Cells pretreated with LPS, IFN-γ, and FAA (50 μM) for 4 h and then with **PIS**; (**g**) Average TPEF intensities in (**a**–**f**), *n* = 5. Scale bar: 20 μm. (Reprinted from reference [32]).

Recently, the imidazoline-2-thione containing OCl^−^ probes, **15** (**PIS**) and **16** (**NIS**), were designed as new fluorescent probes for HOCl (Figure 9) [32]. Upon the addition of up to 5 μM OCl^−^, a new absorbance peak for **PIS** at 378 nm appeared with the sacrifice of the peak at 420 nm in PBS (pH 7.4). Addition of OCl^−^ (0–10 µM) also induced a new fluorescence emissionat 505 nm. We believe the PIS reaction with HOCl generates imidazolium salt **17** and the proposed mechanism is illustrated in Figure 9. **NIS** displayed similar changes with shorter emission wavelengths. To demostrate the possible bio-applications of these probes, **PIS** was used to visualize OCl^−^ generation in RAW 264.7 macrophages, which were activated by lipopolysaccharides (LPS) and then IFN-γ. H_2_O_2_ produced by phorbol myristate acetate (PMA) was transformed to OCl^−^ by MPO. As expected, bright green fluorescence was observed in RAW 264.7 macrophages. When the known MPO inhibitors, 4-aminobenzoicacid hydrazide (ABAH) and flufenamic acid (FFA) were added, distinct fluorescence quenching was observed, which means that **PIS** successfully visualized OCl^−^ production in RAW 264.7 macrophages. We designed a co-culture system of RAW 264.7 macrophages and HeLa cell. When these cell mixtures were treated with stimulants to generate OCl^−^, the macrophages and HeLa cells had distinguishably different shapes and further, green fluorescence was observed only for RAW 264.7 macrophages. Finally, **PIS** was utilized to detect OCl^−^ by using TPM. As shown in Figure 10, under similar conditions of RAW 264.7 macrophage experiments, **PIS** could successfully image OCl^−^ production.

**Figure 11:**
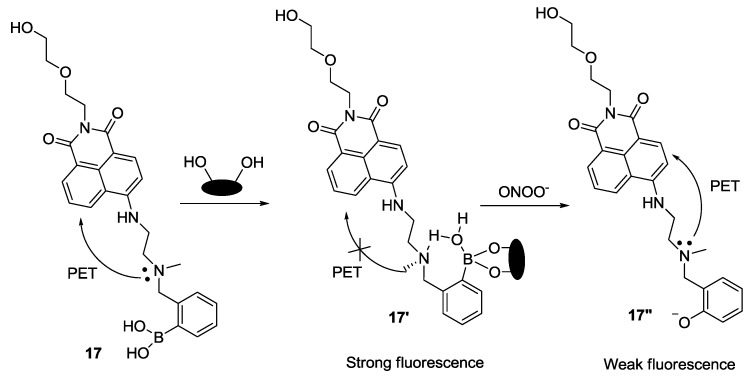
Reaction scheme for probe **17** with ONOO^−^.

James and our group recently reported a unique system for sensing peroxynitrite (ONOO^−^) using boronate-based fluorescent probe **17** and d-fructose (Figure 11) [33]. When d-fructose was added to probe **17** at pH 7.3, a large fluorescence enhancement was observed. Among the various ROS and RNS species, only ONOO^−^ induced a significant fluorescence quenching effect. We believe that the unique interaction of probe **17** with d-fructose plays two important roles in this study, namely to strengthen the fluorescence signal and to prevent the oxidation of boronic acid by other ROS/RNS. This system was also successfully applied to image endogenous and exogenous ONOO^−^ in RAW 264.7 cells and HeLa cells.

**Figure 12:**
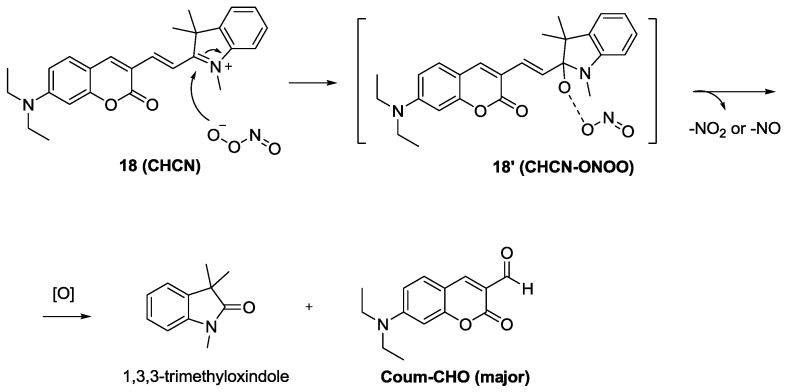
Reaction scheme of peroxinitrite probe **18**.

**Figure 13:**
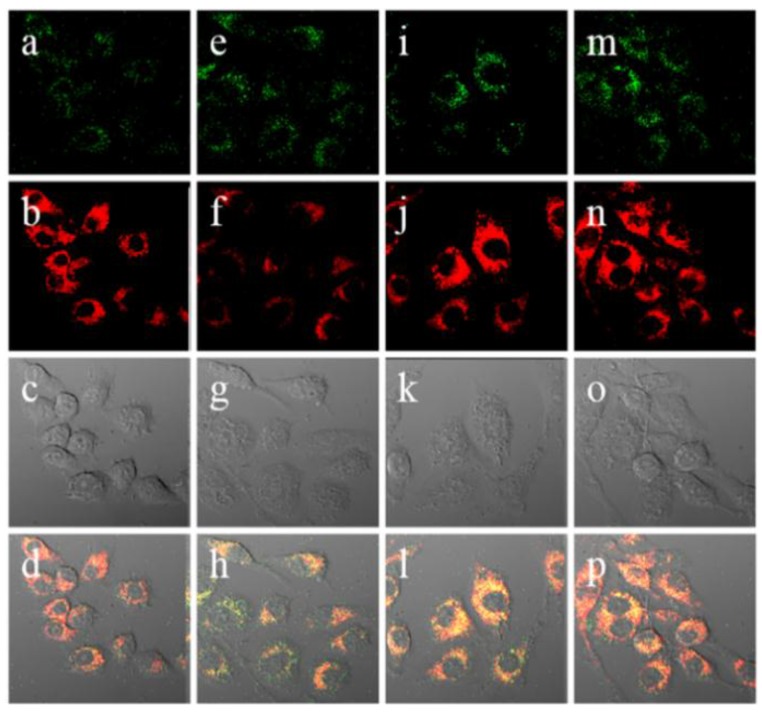
Confocal ratiometric fluorescence images of RAW 264.7 cells for endogenous ONOO^−^ during phagocytic immune response. The cells were stained with 5 μM **18** (**CHCN**) for 30 min and then washed with DPBS before imaging. (**a**) Control; (**e**) lipopolysaccharides (LPS) (1 μg/mL) for 16 h, interferon-γ (50 ng/mL) for 4 h, PMA (10 nM) for 30 min; (**i**) LPS (1 μg/mL) for 16 h, interferon-γ (50 ng/mL) for 4 h, PMA (10 nM) for 30 min, and then AG (1 mM) for 16 h; (**m**) LPS (1 μg/mL) or 16 h, interferon-γ (50 ng/mL) for 4 h, PMA (10 nM) for 30 min, and then TEMPO (100 μM) for 16 h. The green channel (**a**,**e**,**I**,**m**) represents the fluorescence obtained at 490–540 nm with an excitation wavelength at 473 nm, the red channel (**b**,**f**,**j**,**n**) represents the fluorescence obtained at 575–675 nm with an excitation wavelength at 559 nm, images (**c**,**g**,**k**,**o**) represent DIC channels (differential interference contrast), and images (**d**,**h**,**I**,**p**) represent merged images of red and green channels, respectively. (Reprinted from reference [34]).

The red emitting probe **18** (**CHCN**) composed of a linked coumarin-hemicyanine was recently developed as a dual ratiometric and colorimetric probe for peroxynitrite (ONOO^−^) (Figure 12) [34]. Among the various ROS and RNS, only ONOO^−^ induced significant ratiometric fluorescence changes (F_515nm_/F_635nm_). The proposed reaction mechanism is shown in Figure 12. The formation of two products, 1,3,3-trimethyloxindole and Coum-CHO was also confirmed. As shown in Figure 13, **18** (**CHCN**) displayed ratiometric fluorescence changes for exogenous and endogenous ONOO^−^ during the phagocytic immune response. More specifically, red fluorescence was reduced with the enhancement of green fluorescence when RAW 264.7 cells were treated with LPS and IFN-g followed by additional stimulation with PMA. Treatment with a superoxide scavenger, 2,2,6,6-tetramethyl-1-piperidinyloxy (TEMPO) or an NO synthase inhibitor, aminoguanidine, caused no changes in the ratios of red/green fluorescence.

**Figure 14:**
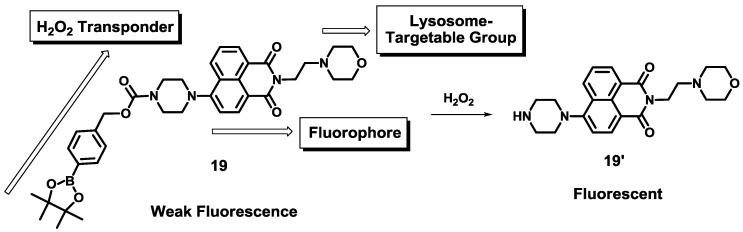
Design strategy of H_2_O_2_ selective probe **19** and its reaction scheme with H_2_O_2_.

Recently, a new boronate-based naphthalimide derivative **19** was developed as a H_2_O_2_ selective fluorescent probe (Figure 14) [35]. Figure 14 explains the design strategy for probe **19**, in which a morpholine moiety acts as a lysosome-targetable group and a *p*-dihydroxyborylbenzyloxycarbonyl group was used as a transponder. Among various ROS and RNS, probe **19** showed a selective fluorescent enhancement at 528 nm at pH 7.4 (0.1 M PBS containing 1% DMF). Probe **19** was clearly localized in the lysosomes, which was confirmed by costaining with LysoTracker Blue DND-22. Probe **19** was further applied to image the level of endogenous and exogenous H_2_O_2_ in the lysosome of the RAW 264.7 cells. Furthermore, time-dependent fluorescence of **19** in the cells was reported.

### 2.3. Fluorescent Imaging Probes for Metal Ions

Among the biologically abundant metal ions, Zn^2+^ is known to play key roles in a variety of physiological processes, such as Alzheimer’s disease, epilepsy, ischemic stroke, *etc*. Due to the lower reactivity of this metal ion, most Zn^2+^ selective fluorescent probes are based on the design of selective ligands and effective fluorescence changes [36].

**Figure 15:**
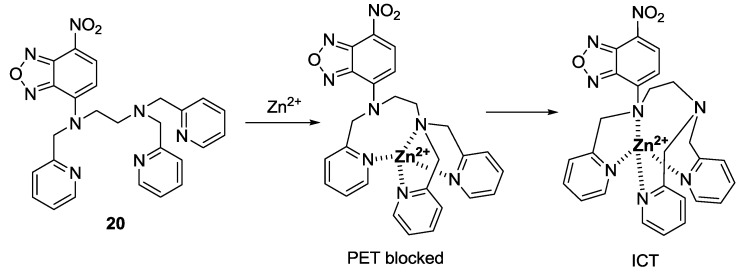
Proposed binding mode of probe **20** with Zn^2+^.

In 2009, we reported a 7-nitrobenz-2-oxa-1,3-diazole (NBD) derivative as a fluorescent and colorimetric probe for Zn^2+^ (Figure 15) [37]. Among the various metal ions, probe **20** displayed a selective fluorescence enhancement only with Zn^2+^ in 100% aqueous solution (0.1 M HEPES, pH 7.2). In addition, red to yellow color change with Zn^2+^ was observed by the naked eye. A large fluorescence enhancement as well as colorimetric changes was attributed to photoinduced electron transfer (PET) and internal charge transfer (ICT) mechanisms as shown in Figure 15. The dissociation constant (*K*_d_) for **20** was reported as 1.3 μM. Relatively high concentrations of Zn^2+^ are present in pancreatic islets, which are known to play a key role in insulin biosynthesis and storage. The practical-use probe **20** could successfully detect the intrinsic Zn^2+^ Ions in pancreatic β-cells.

**Figure 16:**
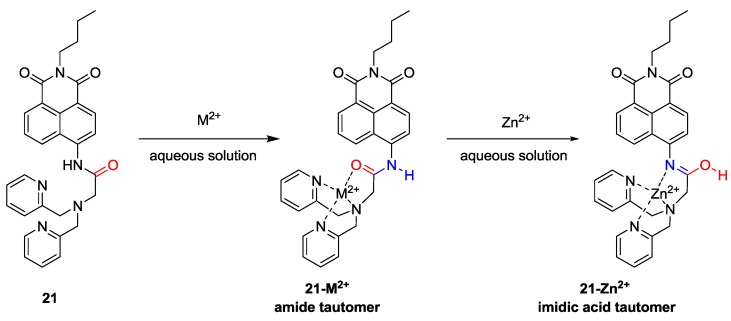
Proposed binding modes of probe **21** with metal ions.

**Figure 17:**
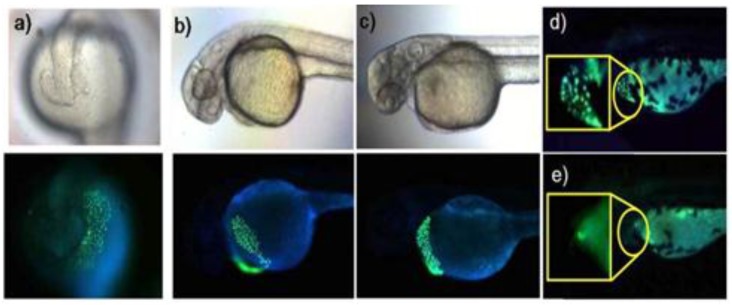
Zebrafish incubated with probe **21** (5 μM). (**a**) Images of 19 h-old; (**b**) 36 h-old; and (**c**) 48 h-old zebrafish incubated with **21** for 1 h; (**d**) Image of 54 h-old zebrafish incubated with **21** for 1 h and (**e**) image of 54 h-old zebrafish after initial incubation with 100 μM TPEN for 1 h and subsequent treatment of washed zebrafish with **21** for 1 h. (Reprinted from reference [38]).

We recently proposed a new strategy called “receptor transformer” [38]. As shown in Figure 16, the naphthalimide derivative (**21**) binds Zn^2+^ in an imidic acid tautomeric form of the probe in aqueous solutions while other heavy transition metal ions, especially Cd^2+^, bind to probe **21** in an amide tautomeric form. With the aid of these unique binding modes, probe **21** displayed a selective fluorescence enhancement for Zn^2+^ over other metal ions with a red-shift from 483 to 514 nm. On the other hand, the addition of Cd^2+^ induced an enhanced blue-shift in emission from 483 to 446 nm in aqueous solution, which binds to probe **21** with an amide tautomeric form. Therefore, green and blue fluorescence was observed for *in vitro* and *in vivo* Zn^2+^ and Cd^2+^, respectively. These different binding modes were confirmed by NMR and IR data. The dissociation constants (*K*_d_) for Zn^2+^ and Cd^2+^ were reported as 5.7 nM and 48.5 nM, respectively. Finally, as shown in Figure 17, **21** could successfully detect intrinsic zinc ions during the development of living zebrafish embryos.

**Figure 18:**
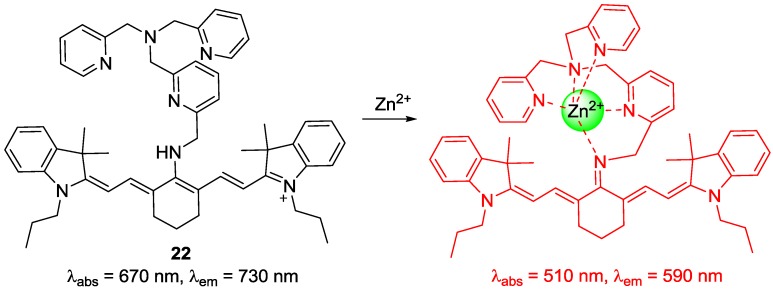
Proposed binding mode of probe **22** with Zn^2+^.

**Figure 19:**
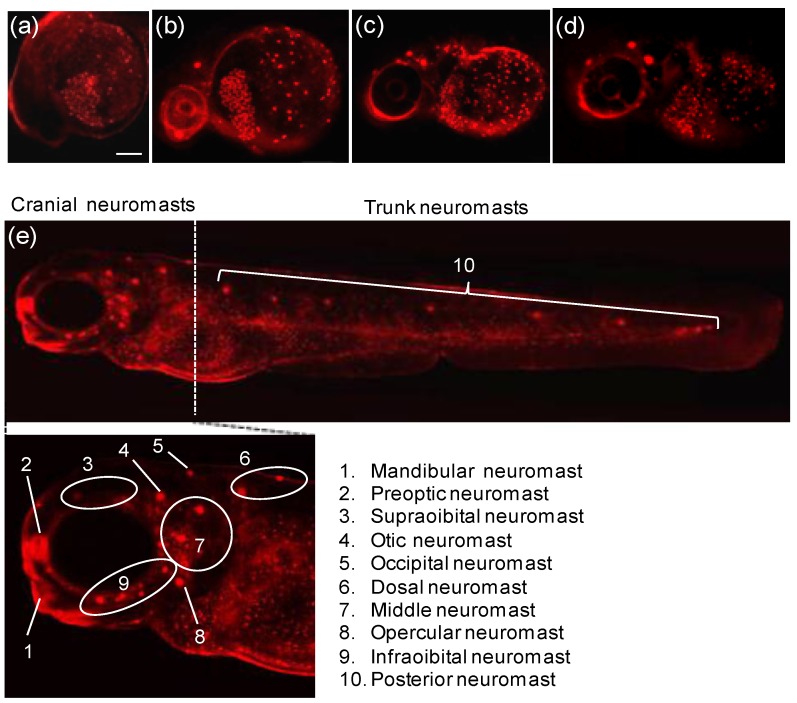
Fluorescent detection of intact zinc ions in zebra fish using probe **22** (**a**) 24 h; (**b**) 36 h; (**c**) 48 h; (**d**) 72 h and (**e**) 96 h-old zebra fish incubated with probe **22** for 1 h. (Reprinted from reference [39]).

A new cyanine-based NIR fluorescent probe **22** bearing tris(2-pyridylmethyl)amine (TMPA) was reported by our group to detect endogenous zinc ions in living cells and organisms (Figure 18) [39,40]. The unique hypsochromic shifts with Zn^2+^ in absorption (670–510 nm) and emission (730–590 nm) were explained as follows: Introduction of Zn^2+^ to the TMPA moiety in **22** deprotonated the amine attached to the center of the polymethine chain of tricarbocyanine to form an imine, which induced the less delocalized diamino-tetraene group. Probe **22** displayed a very strong binding affinity (*K*_d_ = 1.2 nM), which means that probe **22** can serve as an excellent probe to monitor Zn^2+^ in the nanomolar range. Most importantly, probe **22** was used to detect Zn^2+^ released during apoptosis induced by the addition of H_2_O_2_. It is known that metallothioneins, expressed in neuromasts, play a key role in Zn^2+^ homeostasis [41]. Figure 19 clearly shows that probe **22** could detect intact Zn^2+^ in the neuromasts of zebrafish.

Due to the high toxicity of mercury and methyl mercury, the development of new fluorescent probes has been actively explored by many groups [42]. During the last few years, our group has utilized the unique ring-opening process of spirolactam in rhodamine derivatives to develop fluorescent and colorimetric probes for various metal ions in which the ring-opening process of spirolactam can induce large fluorescent enhancements and colorimetric changes (colorless to red) [43,44].

**Figure 20:**
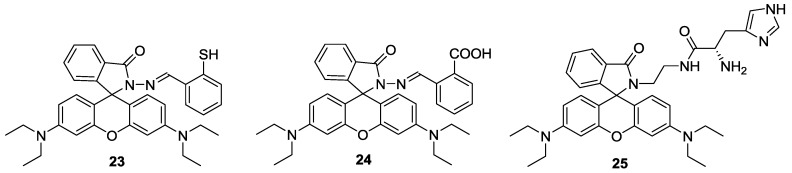
Structures of Hg^2+^ selective probes **23**–**25**.

Two simple rhodamine hydrazone derivatives bearing thiol (**23**) and carboxylic acid (**24**) groups were developed as selective fluorescent and colorimetric probes for Hg^2+^ (Figure 20) [45]. Upon the addition of Hg^2+^, the selective ring-opening process of spirolactams in probes **23** and **24** were observed in CH_3_CN-H_2_O (1:99, *v*/*v*) solution, which was accompanied by large fluorescent enhancement and colorimetric change. The detection limits were calculated as 1 nM for **23** and 4.2 nM for **24**. Both probes could successfully image Hg^2+^ accumulated in the nematode *C. elegans*, which was pretreated with nanomolar concentrations of Hg^2+^.

On the other hand, a rhodamine-based sensor **25** bearing a histidine group was also reported as a selective probe for Hg^2+^ (Figure 20) [46]. Upon the addition of 100 equiv Hg^2+^ in 0.02 M pH 7.4 HEPES: EtOH (1:9, *v*/*v*), turn-on fluorescence (100-fold) was observed due to the spiro-lactam ring opening. We believe that imidazole nitrogen and two carbonyl oxygenscan provided a nice binding site for Hg^2+^. Probe **25** was further applied to visualize Hg^2+^ in HeLa cells.

**Figure 21:**
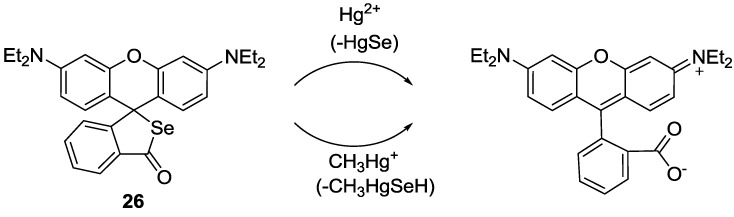
Proposed reaction scheme for probe **26** with Hg^2+^ and CH_3_Hg^+^.

**Figure 22:**
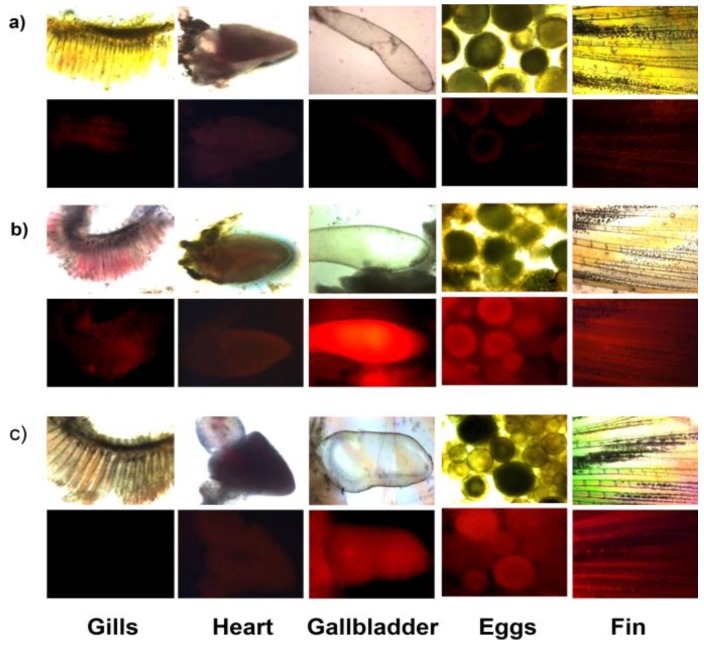
Images of zebrafish organs treated with 20 µM **26** (0.2% DMSO) and 500 nM HgCl_2_ or 500 nM CH_3_HgCl. (**a**) Images of zebrafish organs treated with **26** in the absence of HgCl_2_ or CH_3_HgCl; (**b**) presence of 500 nM HgCl_2_ or (**c**) presence of 500 nM CH_3_HgCl (upper, merged images; lower, fluorescence images). (Reprinted from reference [47]).

A rhodamine derivative bearing the selenolactone group **26** was synthesized for the detection of inorganic and organic mercury species (Figure 21) [47]. Through the mercury ion-promoted deselenation reaction, probe **26** displayed highly selective fluorescent and colorimetric changes for mercury species with high sensitivity in 20 mM HEPES buffer (pH 7.4, 1% CH_3_CN). We then attempted to monitor the mercury species that accumulated in living organisms using **26**. Zebrafish has been used as a suitable animal model for the study of fluorescent probes [48]. For our study, adult zebrafish was pre-incubated with Hg^2+^ (500 nM) or methylmercury (500 nM) and then with probe **26**. As shown in Figure 22, strong red fluorescence was observed in the gallbladder, eggs and fin due to the presence of inorganic mercury and methylmercury.

**Figure 23:**
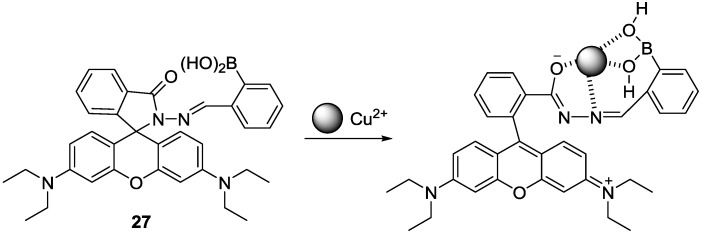
Proposed binding mode of probe **27** with Cu^2+^.

Rhodamine derivative bearing boronic acid (**27**) was studied as a probe for Cu^2+^ for the first time (Figure 23) [49]. When Cu^2+^ was introduced to probe **27** in 20 mM HEPES (0.5% CH_3_CN) at pH 7.4, selective turn-on fluorescence and distinct colorimetric change was observed, which was also attributed to the Cu^2+^-induced spirolactam ring opening process. Probe **27** was applied to visualize Cu^2+^ in cells and zebra fish.

**Figure 24:**
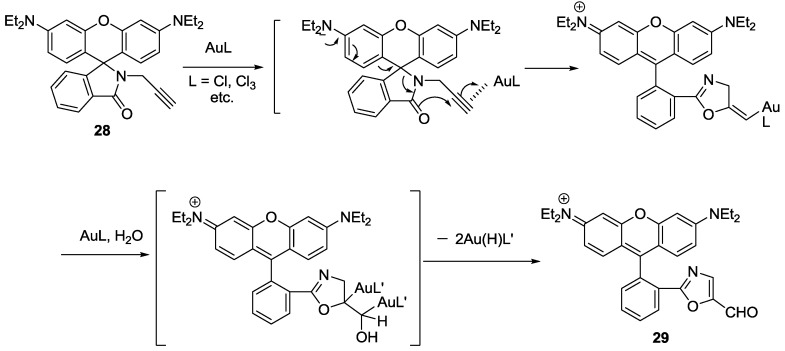
Proposed reaction scheme of probe **28** with Au^3+^.

A reaction based fluorescent probe based on rhodamine-alkyne derivative **28** was synthesized for the detection of Au^3+^ (Figure 24) [50]. The addition of Au^3+^ induced a highly selective turn-on fluorescence and colorimetric change in EtOH-HEPES buffer (0.01 M, pH 7.4) (1:1, *v*/*v*). The proposed reaction mechanism from the propargylamide moiety of **28** to the oxazolecarbaldehyde of **29** is illustrated in Figure 24. The rate constant for the conversion of **28** to **29** was reported to be *K*_obs_ = 4.5 (±0.20) × 10^−4^ s^−1^ and the detection limit was calculated as 320 nM. In addition, probe **28** was successfully applied to detect Au^3+^ in the cell.

**Figure 25:**
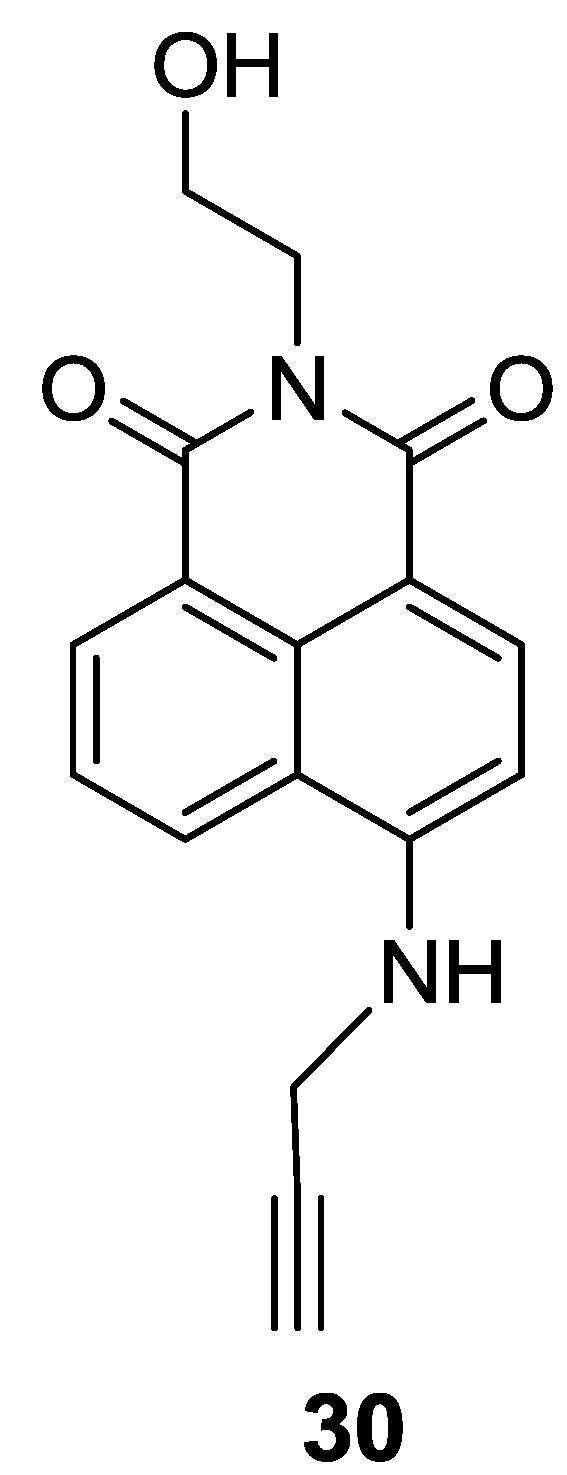
Structure of the probe **30**.

**Figure 26:**
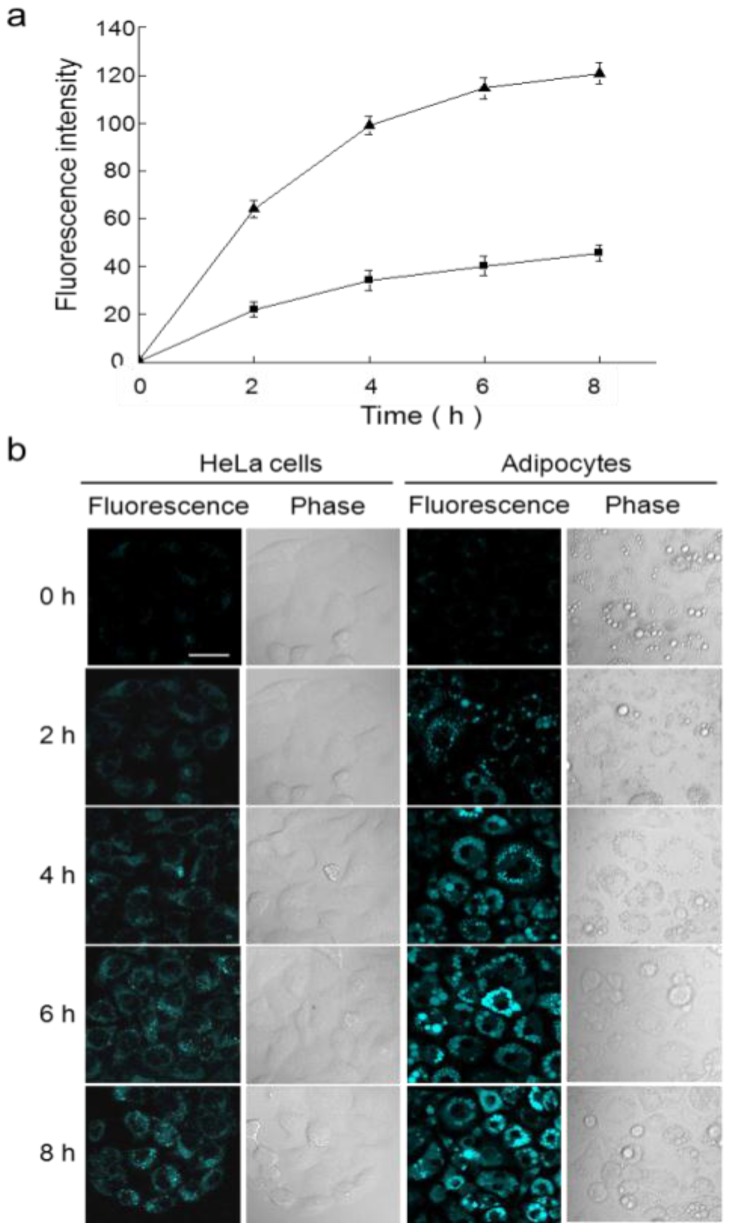
(**a**) Response rates of **30** to Au^3+^ in HeLa cells and differentiated adipocytes. HeLa cells and adipocytes were incubated with 100 μM AuCl_3_ for 2 h and then with **30** (20 μM) for 2, 4, 6, and 8 h (■: HeLa cells, ▲: adipocytes); (**b**) Time-dependent fluorescence images of HeLa cells and adipocytes treated with **30** and Au^3+^ (scale bar = 20 μm). (Reprinted from reference [51]).

A 4-Amino-1,8-naphthalimide derivative bearing an alkyne group (**30**) was explored as a fluorescent probe for Au^3+^ (Figure 25 and Figure 26) [51]. The addition of Au^3+^ induced a distinct color change from yellow to light pink and a large blue shift by about 56 nm in the emission spectra. The detection limit was calculated to be 8.44 μM. We observed that surfactants could enhance the reaction rate of probe **30** with Au^3+^. Probe **30** was also applied to detect Au^3+^ in HeLa cells and differentiated adipocytes. In particular, probe **30** showed a unique ability to detect Au^3+^ in lipid droplets in cells. As shown in Figure 26, the response rates of **30** with Au^3+^ in differentiated adipocytes were greater than those in HeLa cells, which is consistent with the rate enhancement in the presence of surfactants.

### 2.4. Fluorescent Imaging Probes for Cyanide and ATP

Cyanide ion is a toxic anion. Especially, HCN production by *Pseudomonas aeruginosa* (PA) is known to be closely related to the pathogenesis of CF lung disease. Accordingly, the detection of cyanide in *in vivo* systems has become an important issue for bacterial cyanogenesis and CF patients. Cyanide selective fluorescent probes have been actively studied using various design strategies [52].

**Figure 27:**
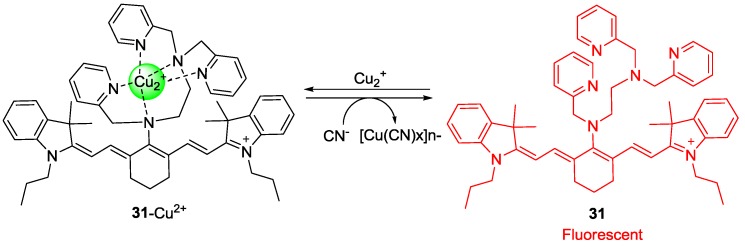
Structure of cyanide selective probe **31** and **31-Cu^2+^** complex.

**Figure 28:**
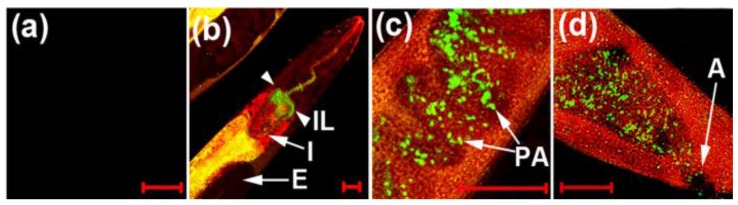
NIR imaging of cyanide in *C. elegans* infected with a *P. aeruginosa* strain (PA14) labeled with green fluorescent protein (GFP). Before the imaging, the nematodes fed on either non-infectious *E. coli* OP50 (**a**) or GFP-labeled PA14 for 2 days (**b**–**d**); (**b**) the anterior end; (**c**) the medial part; (**d**) the posterior end of *C. elegans*. The scale bars represent 20 μm. (IL = intestinal lumen; I = intestine; E = eggs; PA = PA14-GFP; A = anus). (Reprinted from reference [54]).

**Figure 29:**
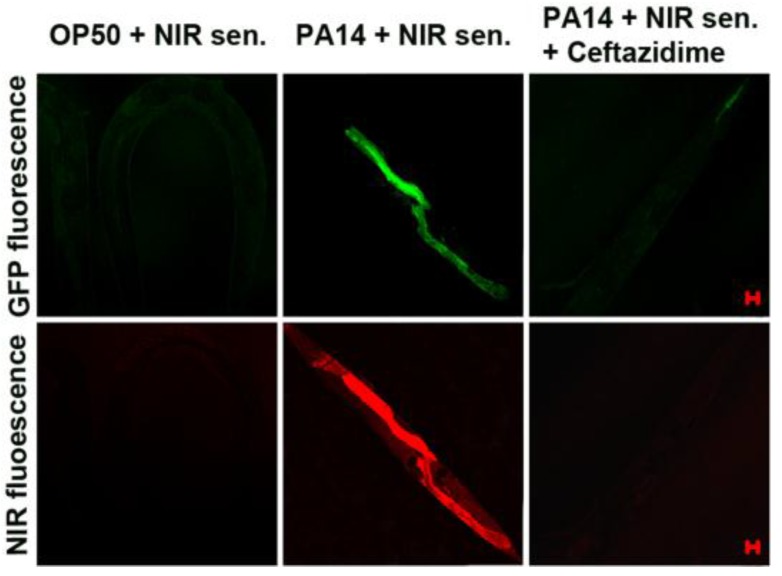
Visualization of antibiotic efficacy against *P. aeruginosa* infection in *C. elegans* with the NIR sensor. The nematodes fed on GFP-labeled *P. aerugionosa* (PA14) for two days. They were then incubated with ceftazidime (200 μg/mL) for 2 h before the *in vivo* imaging. The scale bars represent 20 μm. (Reprinted from reference [53]).

To develop a new cyanide imaging probe, we firstly synthesized NIR fiuorescent probe sensor **31** (Figure 27) [53]. When Cu^2+^ was added and the complex **31-Cu^2+^** was formed, the absorption maximum shifted from 718 to 743 nm and fluorescence quenching (748 nm) was observed in 20 mM HEPES buffer (pH 7.4, 0.5% CH_3_CN). Upon the addition of CN^−^ to **31-Cu^2+^**, fluorescence emission at 748 nm was recovered since CN^−^ and Cu^2+^ form the stable [Cu(CN)_x_]^n−^. The detection limit for CN^−^ was reported as 5 μM. Probe **31-Cu^2+^** was applied to visualize the CN^−^ produced by *P. aeruginosa* (PA) in *C. elegans* cells. To confirm the bacterial infection in the intestine of the nematodes, a PA14 strain labeled with GFP (green fluorescent protein) was introduced for the *C. elegans* to feed on before the imaging. As shown Figure 28a, neither green nor NIR fluorescence was observed in the nematodes after *C. elegans* exposure to only *E. coli* OP50 and probe **31-Cu^2+^**. On the contrary, when nematodes were exposed to PA14 and the probe, both green and NIR fluorescence were observed, which indicates that the probe can image HCN produced by PA14 in the nematodes (Figure 28b–d). As shown in Figure 29, treatment with a β-lactam antibiotic, ceftazidime, significantly reduced both the green and NIR fluorescence intensities.

Imidazolium derivatives serve as host molecules for the recognition of various anions since they can form a unique (C–H)^+^—anion type ionic hydrogen bond [54,55,56].

**Figure 30:**
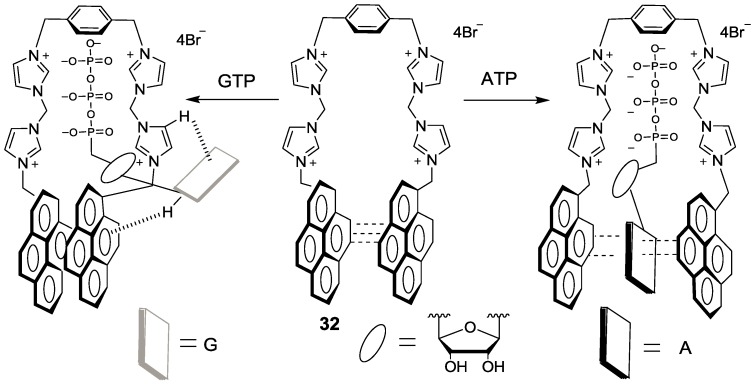
Proposed binding modes of probe **32** with ATP and GTP.

We synthesized an imidazolium receptor bearing two pyrene groups (**32**) as ATP selective probes, in which a pyrene excimer acts as a signal source and imidazoliums serve as the triphosphate anion receptor (Figure 30) [57]. Among the various nucleoside triphosphates, only ATP showed significant ratiometric changes in its emission. More specifically, only ATP induced an increase in monomer emission at 375 with a sacrifice of excimer emission at 487 nm. We believe this result can be attributed to the different binding mode of ATP compared to the other four nucleoside triphosphate bases: GTP, CTP, UTP and TTP. As shown in Figure 30, ATP can form a characteristic sandwich π-π stack of pyrene-adenine-pyrene while GTP, CTP, UTP and TTP interact with probe **32** from the outside, with the stacked pyrene-pyrene dimer of **32** resulting in only excimer fluorescence quenching. These different binding modes were proposed based on NMR study and theoretical calculation results. Oligomycin is reported to decrease cellular ATP levels. Probe **32** showed strong blue fluorescence in HeLa cells and the fluorescence was quenched upon the addition of oligomycin.

## 3. Conclusions and Future Perspectives

Fluorescence is one of the most powerful tools currently available due to its low detection limit and bioimaging capabilities via confocal microscopy. The application of fluorescent imaging probes for various biologically important species has been widely reported in the last decade. In this review, we focused on our exciting contributions in this field. This review was categorized by target analytes, such as biothiols, ROS and RNS, metal ions such as Zn^2+^ and Hg^2+^, and finally cyanide and ATP.

Certainly, their application in biology and environmental science has been a strong driving force for the development of fluorescent probes. We believe that intelligent fluorescent probes will be an important research area in the future, and the next generation of functional fluorescent probes will involve imaging and therapeutic agents *in vivo* [58,59].

## References

[1] Zhou X., Lee S., Xu Z., Yoon J. (2015). Recent Progress on the Development of Chemosensors for Gases. Chem. Rev..

[2] Wu J., Kwon B., Liu W., Anslyn E.V., Wang P., Kim J.S. (2015). Chromogenic/Fluorogenic Ensemble Chemosensing Systems. Chem. Rev..

[3] Shamirian A., Ghai A., Snee P.T. (2015). QD-Based FRET Probes at a Glance. Sensors.

[4] Yin J., Hu Y., Yoon J. (2015). Fluorescence probes and bioimaging: Alkali metals, alkaline earth metals and protons. Chem. Soc. Rev..

[5] Miller E.W., Chang C.J. (2007). Fluorescent probes for nitric oxide and hydrogen peroxide in cell signaling. Curr. Opin. Chem. Biol..

[6] Guo Z., Shin I., Yoon J. (2012). Recognition and Sensing of Various Species Using Boronic Acid Derivatives. Chem. Commun..

[7] Thomas J.A. (2015). Optical imaging probes for biomolecules: An introductory perspective. Chem. Soc. Rev..

[8] Zhou Y., Zhang J.F., Yoon J. (2014). Fluorescent and Colorimetric Chemosensors for Detection of Fluoride Ion. Chem. Rev..

[9] Lee S., Yuen K.K.Y., Jolliffe K.A., Yoon J. (2015). Fluorescent and Colorimetric Chemosensors for Pyrophosphate. Chem. Soc. Rev..

[10] Chen X., Pradhan T., Wang F., Kim J.S., Yoon J. (2012). Fluorescent Chemosensors Based on Spiroring-Opening of Xanthenes and Related Derivatives. Chem. Rev..

[11] Guo Z., Park S., Yoon J., Shin I. (2014). Recent Progress on Near-Infrared Fluorescent Probes for Bioimaging Applications. Chem. Soc. Rev..

[12] Amiot C.L., Xu S., Liang S., Pan L., Zhao J.X. (2008). Near-Infrared Fluorescent Materials for Sensing of Biological Targets. Sensors.

[13] Kim H.M., Cho B.R. (2009). Two-Photon Probes for Intracellular Free Metal Ions, Acidic Vesicles, and Lipid Rafts in Live Tissues. Acc. Chem. Res..

[14] Zhang X., Yin J., Yoon J. (2014). Recent Advances in Development of Chiral Fluorescent and Colorimetric Sensors. Chem. Rev..

[15] De Silva A.P., Gunaratne H.Q.N., Gunnlaugsson T.A., Huxley T.M., McCoy C.P., Rademacher J.T., Rice T.E. (1997). Signaling Recognition Events with Fluorescent Sensors and Switches. Chem. Rev..

[16] Czarnik A.W. (1994). Chemical Communication in Water Using Fluorescent Chemosensors. Acc. Chem. Res..

[17] Yoon J., Czarnik A.W. (1992). Fluorescent Chemosensors of Carbohydrates. A Means of Chemically Communicating the Binding of Polyols in Water Based on Chelation-Enhanced Quenching. J. Am. Chem. Soc..

[18] Tang Y., Lee D., Wang J., Li G., Lin W., Yoon J. (2015). Development of fluorescent probes based on protection-deprotection of aldehyde, hydroxyl, and amino functional groups for biological imaging. Chem. Soc. Rev..

[19] Jun M.E., Roy B., Ahn K.H. (2011). Turn-on fluorescent sensing with reactive probes. Chem. Commun..

[20] Yeung M.C.-L., Yam V.W.-W. (2015). Luminescent cation sensors: From host-guest chemistry, supramolecular chemistry to reaction-based mechanisms. Chem. Soc. Rev..

[21] Jung H.S., Chen X., Kim J.S., Yoon J. (2013). Recent progress in luminescent and colorimetric chemosensors for detection of thiols. Chem. Soc. Rev..

[22] Chen X., Ko S.-K., Kim M.J., Shin I., Yoon J. (2010). A Thiol-Specific Fluorescent Probe and Its Application for Bioimaging. Chem. Commun..

[23] Yang X., Guo Y., Strongin R.M. (2011). Conjugate Addition/Cyclization Sequence Enables Selective and Simultaneous Fluorescence Detection of Cysteine and Homocysteine. Angew. Chem. Int. Ed..

[24] Guo Z., Nam S.W., Park S., Yoon J. (2012). A Highly Selective Ratiometric Near-Infrared Fluorescent Cyanine Sensor for Cysteine with Remarkable Shift and Its Application Bioimaging. Chem. Sci..

[25] Lee H.Y., Choi Y.P., Kim S.K., Yoon T., Guo Z., Swamy K.M.K., Kim G., Lee J.Y., Shin I., Yoon J. (2014). Selective Homocysteine Turn-on Fluorescent Probes and Their Bioimaging Applications. Chem. Commun..

[26] Hu Y., Heo C.H., Kim G., Jun E.J., Yin J., Kim H.M., Yoon J. (2015). One-Photon and Two-Photon Sensing of Biothiols Using A Bis-Pyrene-Cu(II) Ensemble and Its Application to Image GSH in the Cells and Tissues. Anal. Chem..

[27] Yin J., Kwon Y., Kim D., Lee D., Kim G., Hu Y., Ryu J.-H., Yoon J. (2014). A Cyanine Based Fluorescence Probe for Highly Selective Detection of Glutathione in Cell Cultures and Live Mice Tissues. J. Am. Chem. Soc..

[28] Lee D., Kim G., Yin J., Yoon J. (2015). Aryl-thioether substituted nitrobenzothiadiazole probe for selective detection of cysteine and homocysteine. Chem. Commun..

[29] Chen X., Tian X., Shin I., Yoon J. (2011). Fluorescent and luminescent probes for detection of reactive oxygen and nitrogen species. Chem. Soc. Rev..

[30] Chen X., Lee K.-A., Ha E.-M., Lee K.M., Seo Y.Y., Choi H.K., Kim H.N., Kim M.J., Cho C.-S., Lee S.Y. (2011). A specific and sensitive method for detection of hypochlorous acid for the imaging of microbe-induced HOCl production. Chem. Commun..

[31] Xu Q., Lee K.-A., Lee S., Lee K.M., Lee W.-J., Yoon J. (2013). A Highly Specific Fluorescent Probe for Hypochlorous Acid and Its Application in Imaging Microbe-Induced HOCl Production. J. Am. Chem. Soc..

[32] Xu Q., Heo C.H., Kim G., Lee H.W., Kim H.M., Yoon J. (2015). Development of Imidazoline-2-Thiones Based Two-Photon Fluorescence Probes for Imaging Hypochlorite Generation in a Co-Culture System. Angew. Chem. Int. Ed..

[33] Sun X., Xu Q., Kim G., Flower S.E., Lowe J.P., Yoon J., Fossey J.S., Qian X., Bull S.D., James T.D. (2014). A water-soluble boronate-based fluorescent probe for the selective detection of peroxynitrite and imaging in living cells. Chem. Sci..

[34] Zhou X., Kwon Y., Kim G., Ryu J.-H., Yoon J. (2015). A ratiometric fluorescent probe based on a coumarin-hemicyanine scaffold for sensitive and selective detection of endogenous peroxynitrite. Biosens. Bioelectron..

[35] Kim D., Kim G., Nam S.-J., Yin J., Yoon J. (2015). Visualization of Endogenous and Exogenous Hydrogen Peroxide Using A Lysosome-Targetable Fluorescent Probe. Sci. Rep..

[36] Xu Z., Yoon J., Spring D.R. (2010). Fluorescent Chemosensors for Zn^2+^. Chem. Soc. Rev..

[37] Xu Z., Kim G.-H., Han S.J., Jou M.J., Lee C., Shin I., Yoon J. (2009). An NBD-based colorimetric and fluorescent chemosensor for Zn^2+^ and its use for detection of intracellular zinc ions. Tetrahedron.

[38] Xu Z., Baek K.-H., Kim H.N., Cui J., Qian X., Spring D.R., Shin I., Yoon J. (2010). Zn^2+^-Triggered Amide Tautomerization Produces a Highly Zn^2+^-Selective, Cell–Permeable and Ratiometric Fluorescent Sensor. J. Am. Chem. Soc..

[39] Guo Z., Kim G.-H., Shin I., Yoon J. (2012). A Cyanine-Based Fluorescent Sensor for Detecting Endogenous Zinc Ions in Live Cells and Organisms. Biomaterials.

[40] Guo Z., Kim G.-H., Yoon J., Shin I. (2014). Synthesis of a highly Zn^2+^-selective cyanine-based probe and its use for tracing endogenous zinc ions in cells and organisms. Nat. Protocol..

[41] Palmiter R.D. (1998). The elusive function of metallothioneins. Proc. Natl. Acad. Sci. USA.

[42] Kim H.N., Ren W.X., Kim J.S., Yoon J. (2012). Fluorescent and Colorimetric Sensors for Detection of Lead, Cadmium, and Mercury Ions. Chem. Soc. Rev..

[43] Park S., Kim W., Swamy K.M.K., Jung J.Y., Kim G., Kim Y., Kim S.-J., Yoon J. (2013). Rhodamine Hydrazone Derivatives Bearing Thiophene Group as Fluorescent and Colorimetric Chemosensors for Hg^2+^. Dyes Pigm..

[44] Kwon J.Y., Jang Y.J., Lee Y.J., Kim K.-M., Seo M.-S., Nam W., Yoon J. (2005). A Highly Selective Fluorescent Chemosensor for Pb^2+^. J. Am. Chem. Soc..

[45] Kim H.N., Nam S.-W., Swamy K.M.K., Jin Y., Chen X., Kim Y., Kim S.-J., Park S., Yoon J. (2011). Rhodamine Hydrazone Derivatives as Hg^2+^ Selective Fluorescent and Colorimetric Chemosensors and Their Applications to Bioimaging and Microfluidic System. Analyst.

[46] Kwon S.K., Kim H.N., Rho J.H., Swamy K.M.K., Shanthakumar S.M., Yoon J. (2009). Rhodamine Derivative Bearing Histidine Binding Site as a Fluorescent Chemosensor for Hg^2+^. Bull. Korean Chem. Soc..

[47] Chen X., Baek K.-H., Kim Y., Kim S.-J., Shin I., Yoon J. (2010). A Selenolactone-Based Fluorescent Chemodosimeter to Monitor Mercury/ Methylmercury Species *in vitro* and *in vivo*. Tetrahedron.

[48] Ko S.-K., Chen X., Yoon J., Shin I. (2011). Zebrafish as a good vertebrate model for molecular imaging using fluorescent probes. Chem. Soc. Rev..

[49] Swamy K.M.K., Ko S.-K., Kwon S.K., Lee H.N., Mao C., Kim J.-M., Lee K.-H., Kim J., Shin I., Yoon J. (2008). Boronic Acid-Linked Fluorescent and Colorimetric Probes for Copper Ions. Chem. Commun..

[50] Jou M.J., Chen X., Swamy K.M.K., Kim H.N., Kim H.-J., Lee S.-G., Yoon J. (2009). Highly Selective Fluorescent Probe for Au^3+^ Based on Cyclization of Propargylamide. Chem. Commun..

[51] Choi J.Y., Kim G.-H., Guo Z., Swamy K.M.K., Lee H.Y., Pai J., Shin S., Shin I., Yoon J. (2013). Highly Selective Ratiometric Fluorescent Probe for Au^3+^ and Its Application to Bioimaging. Biosens. Bioelectron..

[52] Wang F., Wang L., Chen X., Yoon J. (2014). Recent progress in the development of fluorometric and colorimetric chemosensors for detection of cyanide ion. Chem. Soc. Rev..

[53] Chen X., Nam S.-W., Kim G.-H., Song N., Jeong Y., Shin I., Kim S.K., Kim J., Park S., Yoon J. (2010). A Near-Infrared Fluorescent Sensor for Detection of Cyanide in Aqueous Solution and Its Application for Bioimaging. Chem. Commun..

[54] Xu Z., Kim S.K., Yoon J. (2010). Revisit to Imidazolium Receptors for the Recognition of Anions: Highlighted Research During 2006–2009. Chem. Soc. Rev..

[55] Kim S.K., Singh N.J., Kwon J., Hwang I.-C., Park S.J., Kim K.S., Yoon J. (2006). Fluorescent Imidazolium Receptors for the Recognition of Pyrophosphate. Tetrahedron.

[56] Kim S.K., Kang B.-G., Koh H.S., Yoon Y.J., Jung S.J., Jeong B., Lee K.-D., Yoon J. (2004). A New ImidazoliumCavitand for the Recognition of Dicarboxylates. Org. Lett..

[57] Xu Z., Singh N.J., Lim J., Pan J., Kim H.N., Park S., Kim K.S., Yoon J. (2009). Unique Sandwich Stacking of Pyrene-Adenine-Pyrene for Selective and Ratiometric Fluorescent Sensing of ATP at Physiological pH. J. Am. Chem. Soc..

[58] Kolemen S., Işık M., Kim G.M., Kim D., Geng H., Buyuktemiz M., Karatas T., Zhang X.-F., Dede Y., Yoon J. (2015). Intracellular Modulation of Excited-State Dynamics in a Chromophore Dyad: Differential Enhancement of Photocytotoxicity Targeting Cancer Cells. Angew. Chem. Int. Ed..

[59] Kim E.-J., Bhuniya S., Lee H., Kim H.M., Cheong C., Maiti S., Hong K.S., Kim J.S. (2014). An Activatable Prodrug for the Treatment of Metastatic Tumor. J. Am. Chem. Soc..

